# Supplementary material for: Boosting Single-Cell RNA Sequencing Analysis with Simple Neural Attention
Source: bioRxiv. 2023 Jun 1:2023.05.29.542760. Preprint. [Version 1] doi: 10.1101/2023.05.29.542760 (PMC10312486; doi:10.1101/2023.05.29.542760)
Supplement: 1 [file NIHPP2023.05.29.542760V1-supplement-1.pdf]

bioRxiv preprint doi: https://doi.org/10.1101/2023.05.29.542760; this version posted June 1, 2023. The copyright holder for this preprint (which was not certified by peer review) is the author/funder, who has granted bioRxiv a license to display the preprint in perpetuity. It is made available under aCC-BY 4.0 International license.

Extended Figures

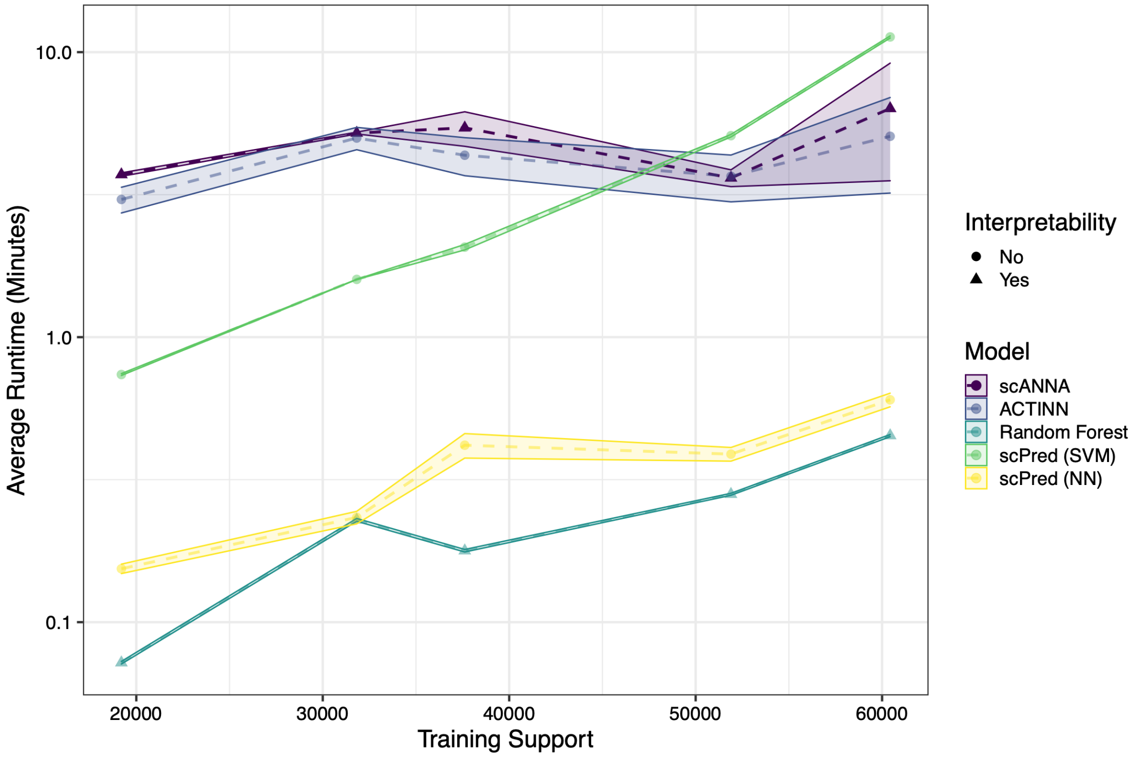

Figure S1. Comparison of average model training times when utilizing GPUs for deep learning models. Although scANNA is a much larger model than ACTINN (∼760M parameters compared to ACTINN's 25M), scANNA trains in a comparable time as ACTINN. Note that Random Forest and scPred were trained on CPUs (same runtimes as Extended Figure 2) and are used here as baselines.

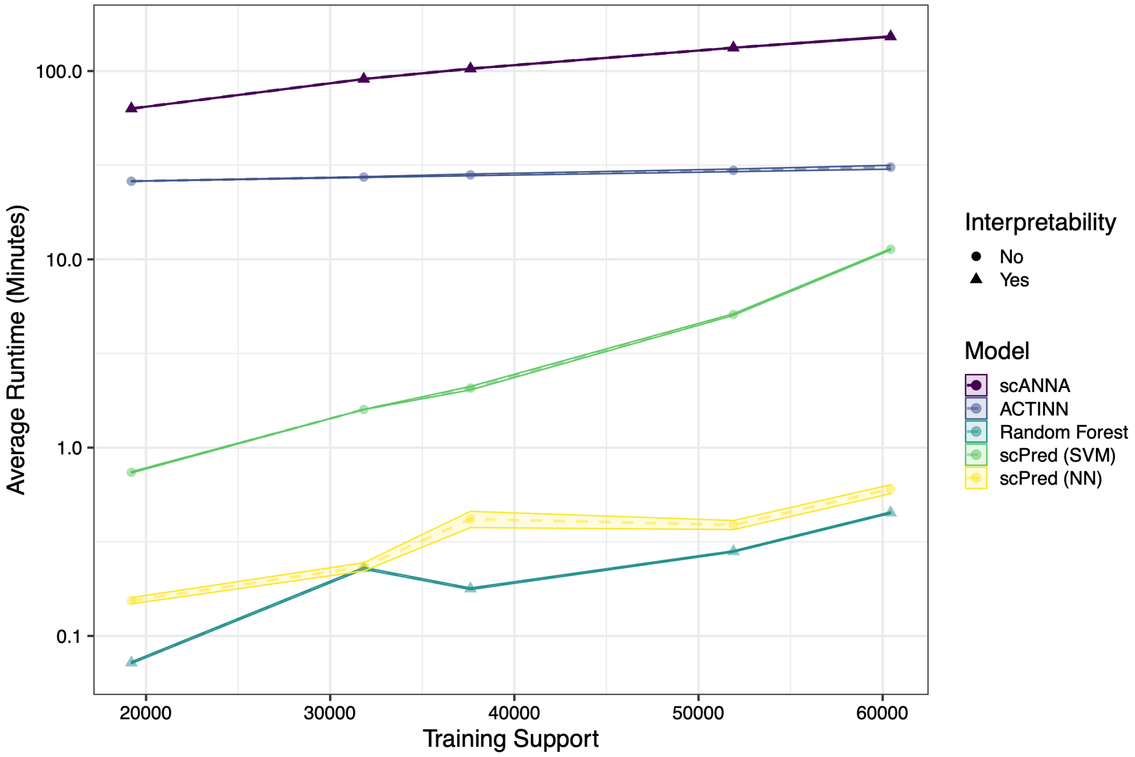

Figure S2. Comparison of average model training times when utilizing CPUs. ScANNA takes considerably longer time to train on a standard computer (provide specifications here) than traditional methods. However, once trained, scANNA can be quickly finetuned on other datasets for downstream analyses (10× reduction in runtime on average).

bioRxiv preprint doi: https://doi.org/10.1101/2023.05.29.542760; this version posted June 1, 2023. The copyright holder for this preprint (which was not certified by peer review) is the author/funder, who has granted bioRxiv a license to display the preprint in perpetuity. It is made available under aCC-BY 4.0 International license.

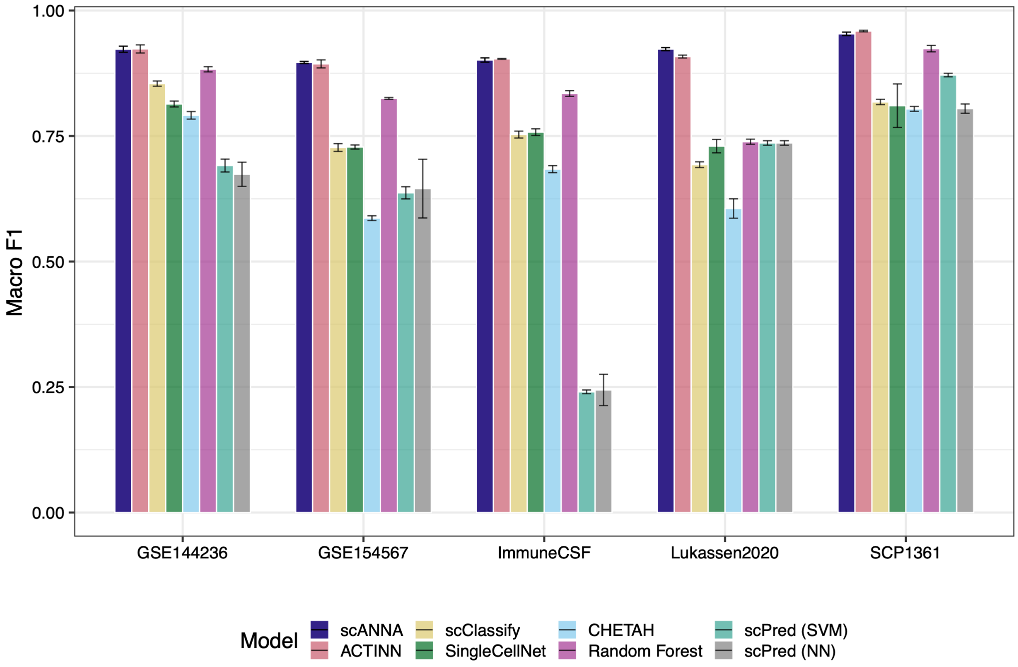

Figure S3. Accuracy of various supervised annotation methods reported in macro F1 score.

bioRxiv preprint doi: https://doi.org/10.1101/2023.05.29.542760; this version posted June 1, 2023. The copyright holder for this preprint (which was not certified by peer review) is the author/funder, who has granted bioRxiv a license to display the preprint in perpetuity. It is made available under aCC-BY 4.0 International license.

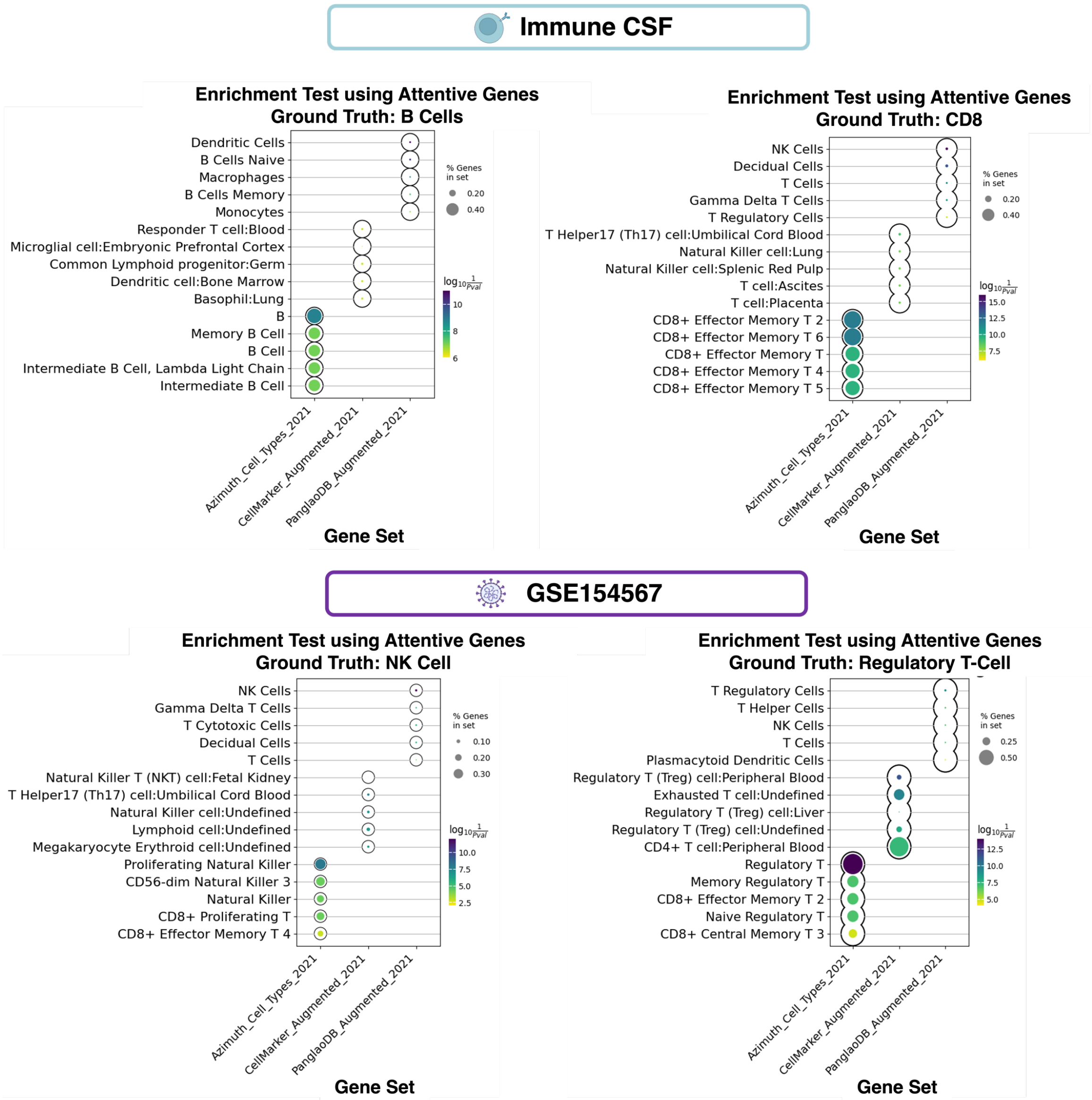

Figure S4. ScANNA's unsupervised broad annotation example (enrichment of local marker genes identified by scANNA).

bioRxiv preprint doi: https://doi.org/10.1101/2023.05.29.542760; this version posted June 1, 2023. The copyright holder for this preprint (which was not certified by peer review) is the author/funder, who has granted bioRxiv a license to display the preprint in perpetuity. It is made available under aCC-BY 4.0 International license.

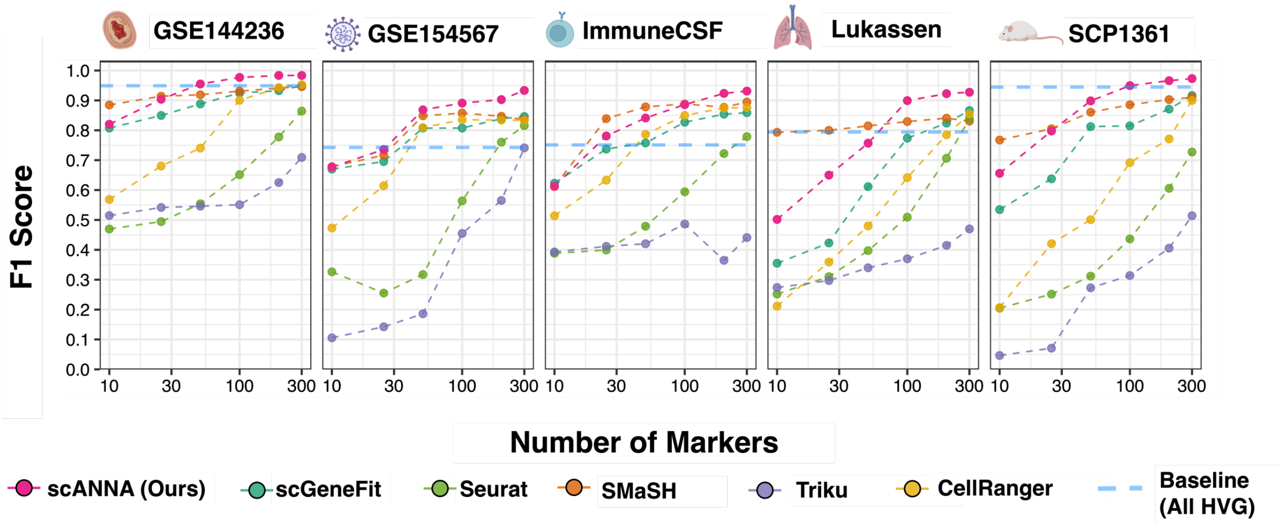

Figure S5. Evaluating global marker selection performance through measuring classification accuracy of cell populations with n selected markers from each model.

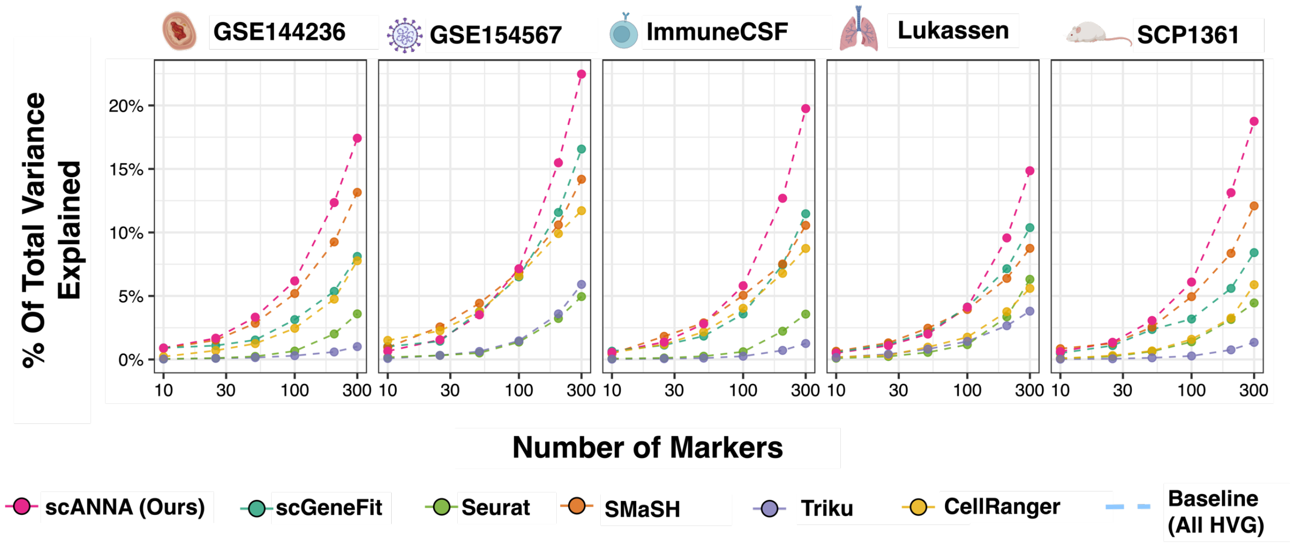

Figure S6. Evaluating global marker selection performance through analyzing the fraction of total variance explained with n selected markers from each model.

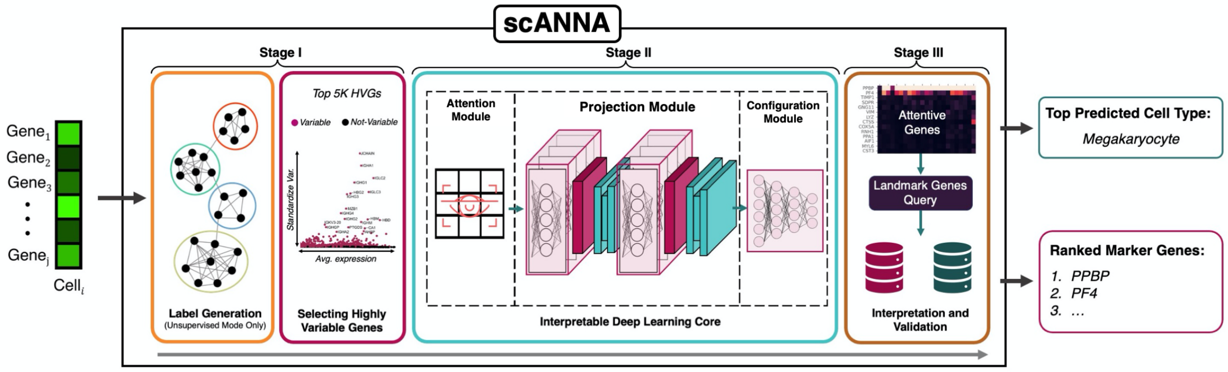

Figure S7. Overview of scANNA's workflow of performing unsupervised annotation used in this study.

bioRxiv preprint doi: https://doi.org/10.1101/2023.05.29.542760; this version posted June 1, 2023. The copyright holder for this preprint (which was not certified by peer review) is the author/funder, who has granted bioRxiv a license to display the preprint in perpetuity. It is made available under aCC-BY 4.0 International license.

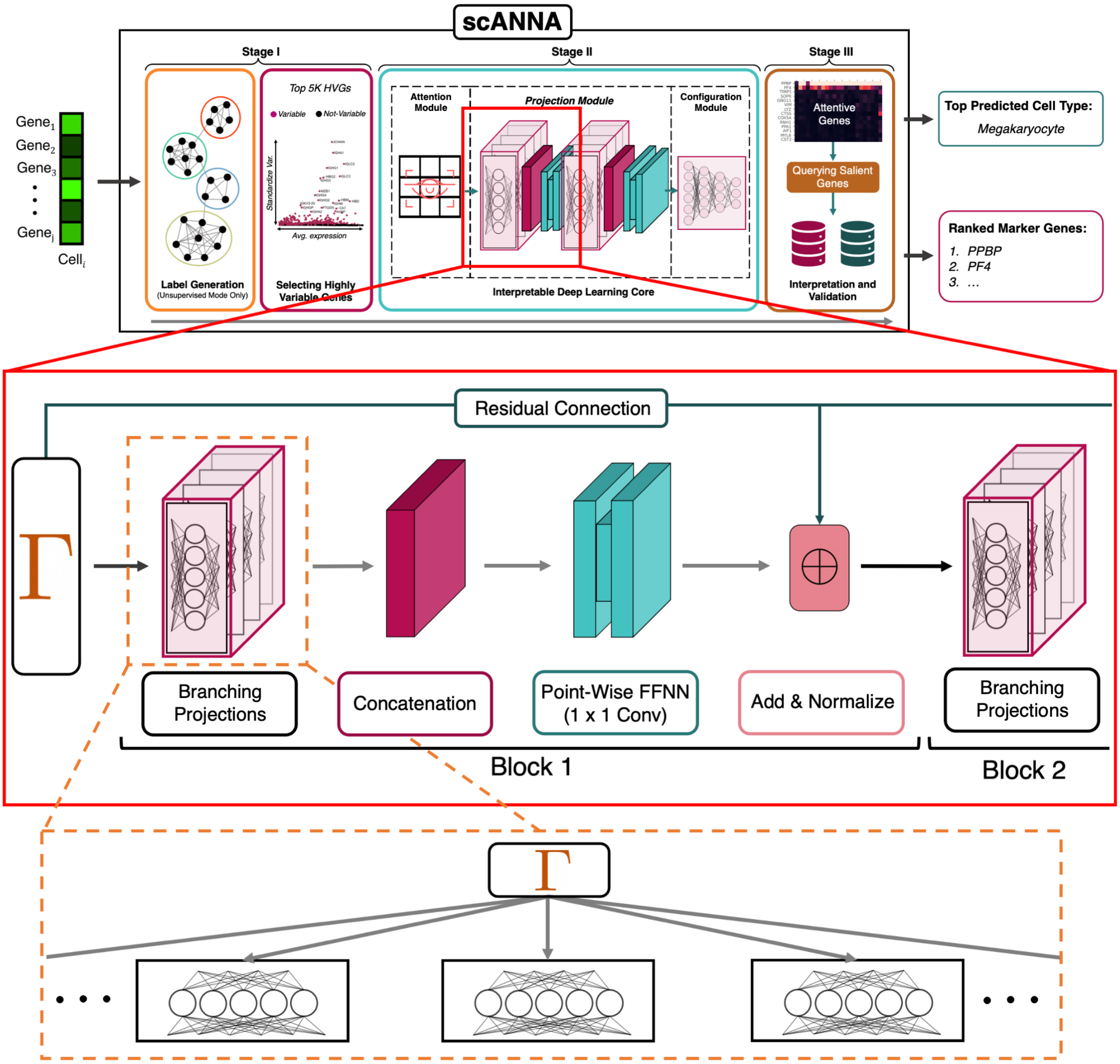

Figure S8. Overview of scANNA's projection block with respect to other components (using the unsupervised annotation as an example).
